# Supplementary material for: Expression Profiling of Candidate Genes in Sugar Beet Leaves Treated with Leonardite-Based Biostimulant
Source: High Throughput. 2019 Oct 11;8(4):18. doi: 10.3390/ht8040018 (PMC6970231; doi:10.3390/ht8040018)
Supplement: Supplementary file 1 [file high-throughput-08-00018-s001.pdf]

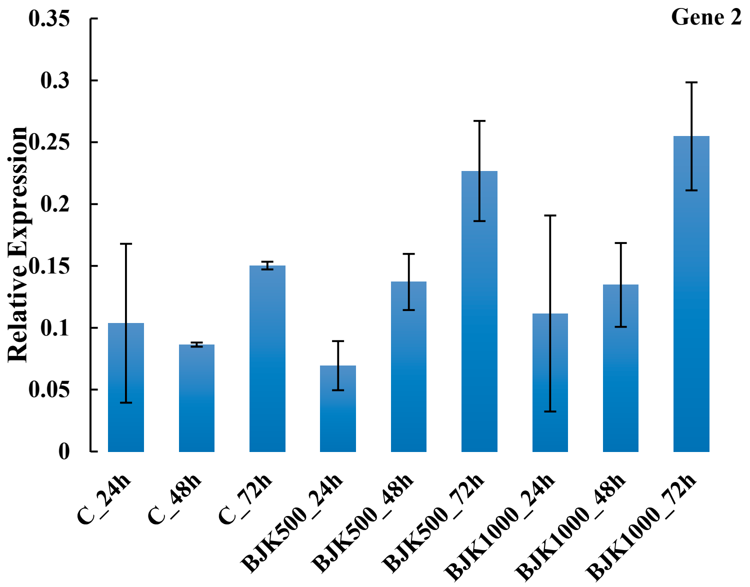
Gene 2
Relative Expression
0.35
0.3
0.25
0.2
0.15
0.1
0.05
0
C_24h
C_48h
C_72h
BJK500_24h
BJK500_48h
BJK500_72h
BJK1000_24h
BJK1000_48h
BJK1000_72h

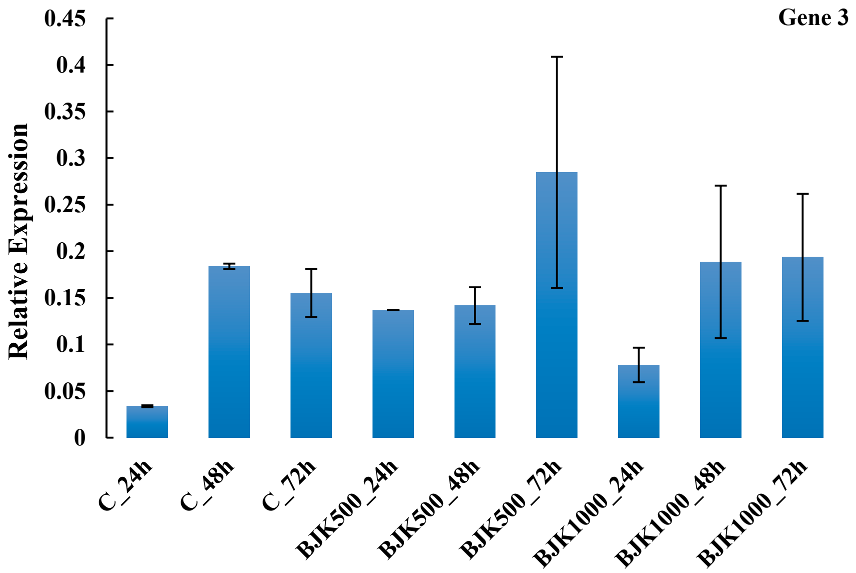
Gene 3
Relative Expression
0.45
0.4
0.35
0.3
0.25
0.2
0.15
0.1
0.05
0
C_24h
C_48h
C_72h
BJK500_24h
BJK500_48h
BJK500_72h
BJK1000_24h
BJK1000_48h
BJK1000_72h

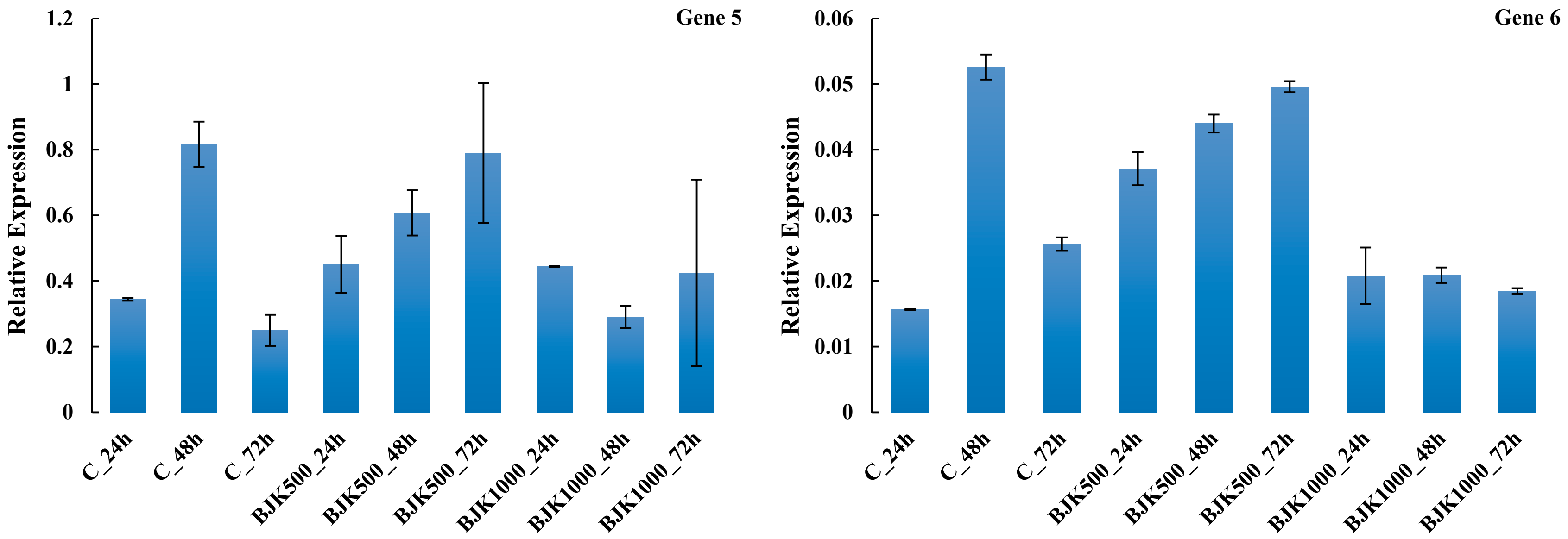
Gene 5
Relative Expression
1.2
1
0.8
0.6
0.4
0.2
0
C_24h
C_48h
C_72h
BJK500_24h
BJK500_48h
BJK500_72h
BJK1000_24h
BJK1000_48h
BJK1000_72h
Gene 6
Relative Expression
0.06
0.05
0.04
0.03
0.02
0.01
0
C_24h
C_48h
C_72h
BJK500_24h
BJK500_48h
BJK500_72h
BJK1000_24h
BJK1000_48h
BJK1000_72h

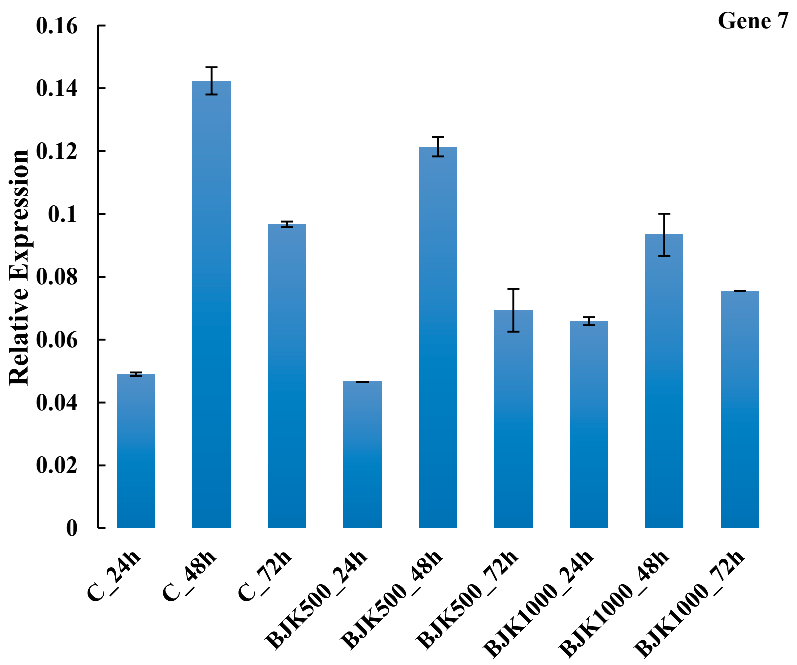

Gene 7
Relative Expression
0.16
0.14
0.12
0.1
0.08
0.06
0.04
0.02
0
C_24h
C_48h
C_72h
BJK500_24h
BJK500_48h
BJK500_72h
BJK1000_24h
BJK1000_48h
BJK1000_72h

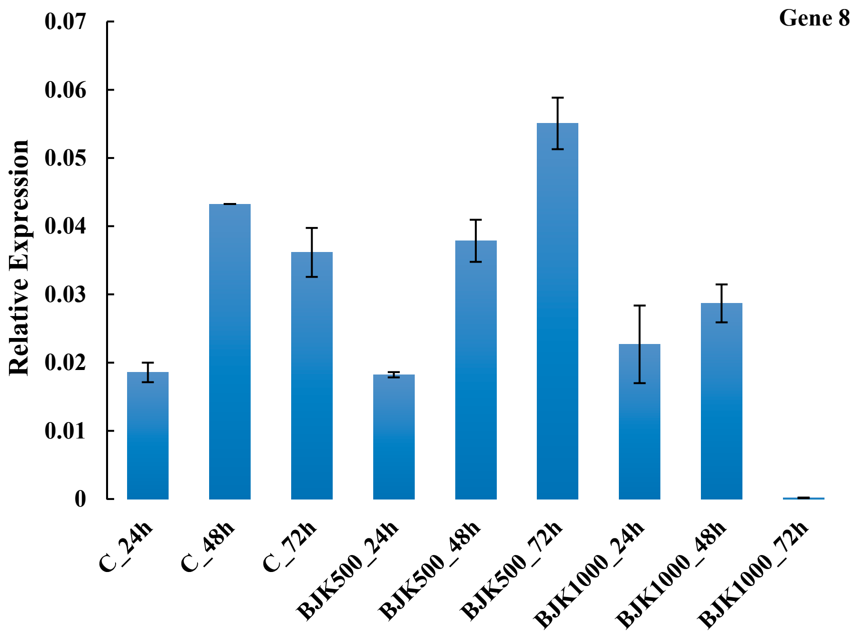

Gene 8
Relative Expression
0.07
0.06
0.05
0.04
0.03
0.02
0.01
0
C_24h
C_48h
C_72h
BJK500_24h
BJK500_48h
BJK500_72h
BJK1000_24h
BJK1000_48h
BJK1000_72h

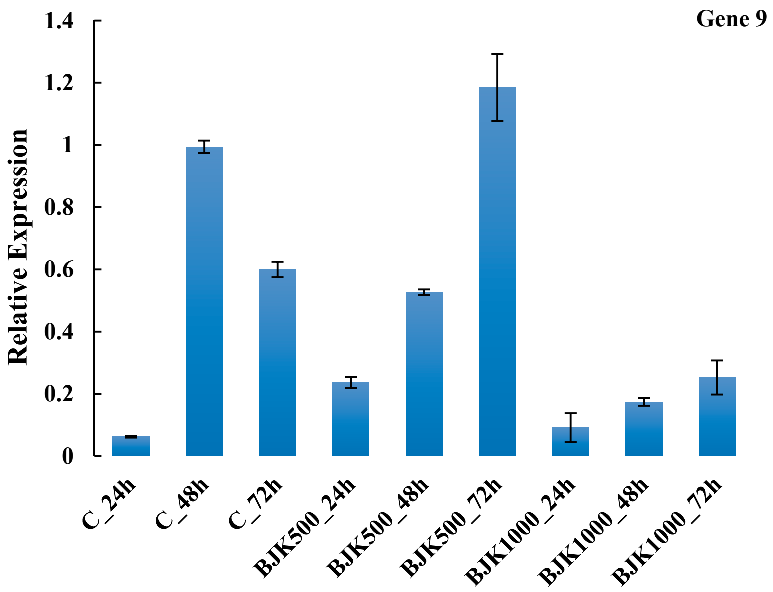
Gene 9
Relative Expression
1.4
1.2
1
0.8
0.6
0.4
0.2
0
C_24h
C_48h
C_72h
BJK500_24h
BJK500_48h
BJK500_72h
BJK1000_24h
BJK1000_48h
BJK1000_72h

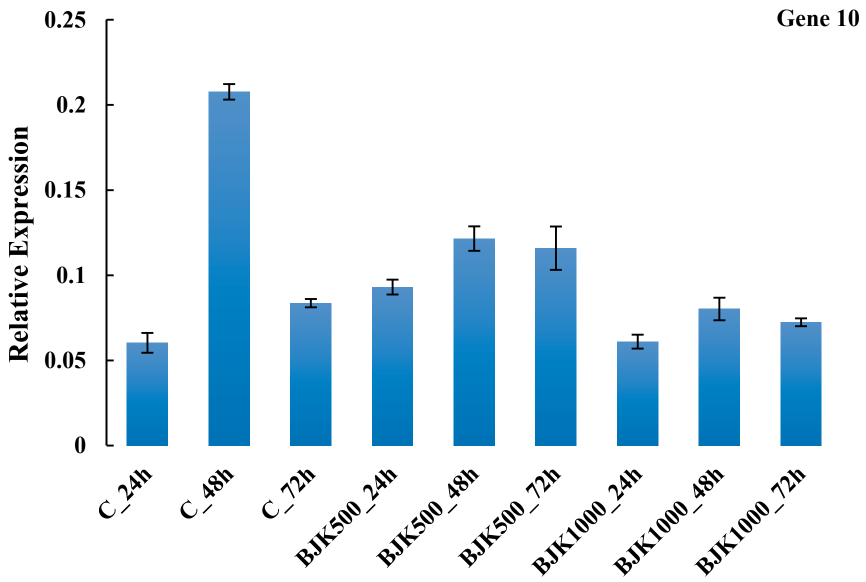
Gene 10
Relative Expression
0.25
0.2
0.15
0.1
0.05
0
C_24h
C_48h
C_72h
BJK500_24h
BJK500_48h
BJK500_72h
BJK1000_24h
BJK1000_48h
BJK1000_72h

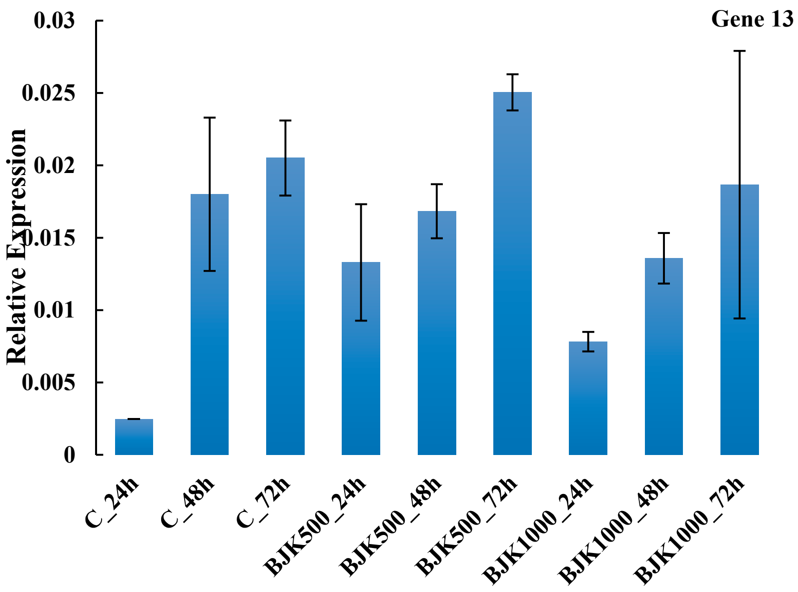
Gene 13
Relative Expression
0.03
0.025
0.02
0.015
0.01
0.005
0
C_24h
C_48h
C_72h
BJK500_24h
BJK500_48h
BJK500_72h
BJK1000_24h
BJK1000_48h
BJK1000_72h

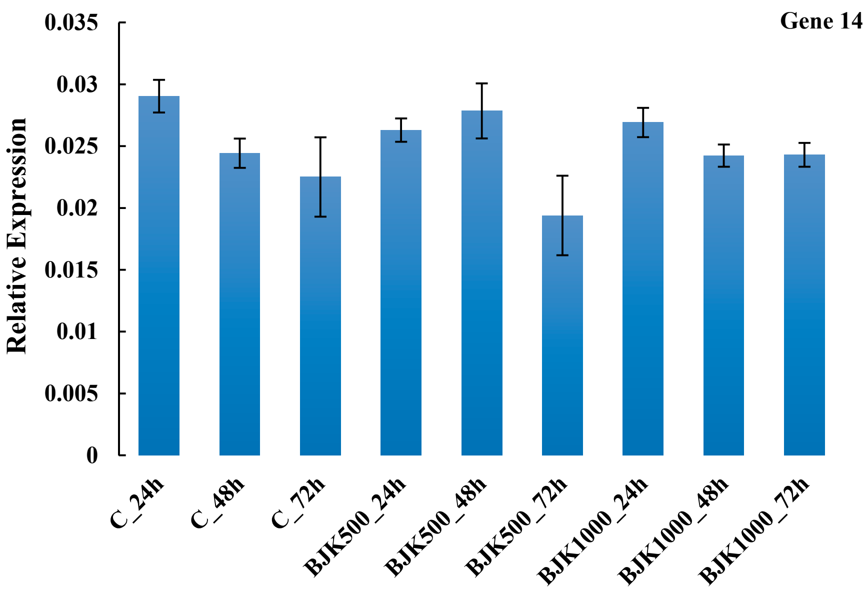
Gene 14
Relative Expression
0.035
0.03
0.025
0.02
0.015
0.01
0.005
0
C_24h
C_48h
C_72h
BJK500_24h
BJK500_48h
BJK500_72h
BJK1000_24h
BJK1000_48h
BJK1000_72h

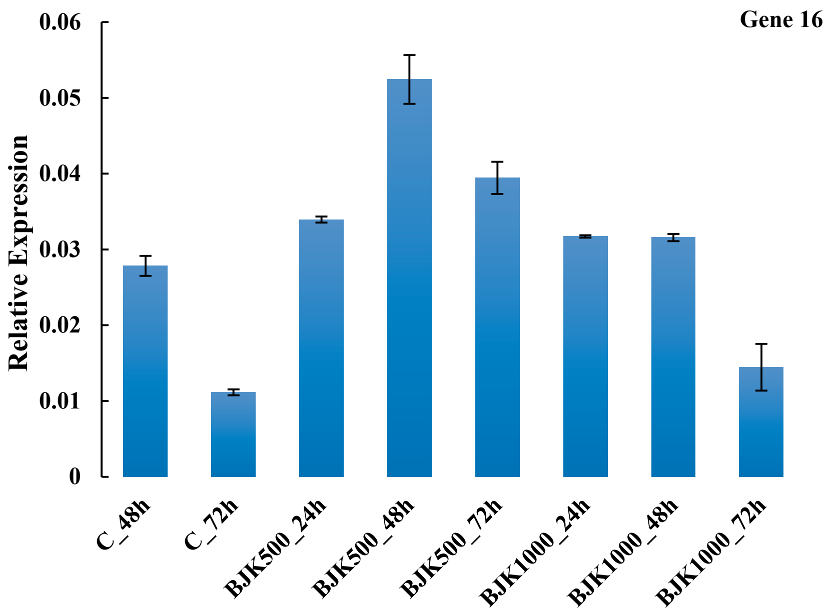
Gene 16
Relative Expression
0.06
0.05
0.04
0.03
0.02
0.01
0
C_48h
C_72h
BJK500_24h
BJK500_48h
BJK500_72h
BJK1000_24h
BJK1000_48h
BJK1000_72h

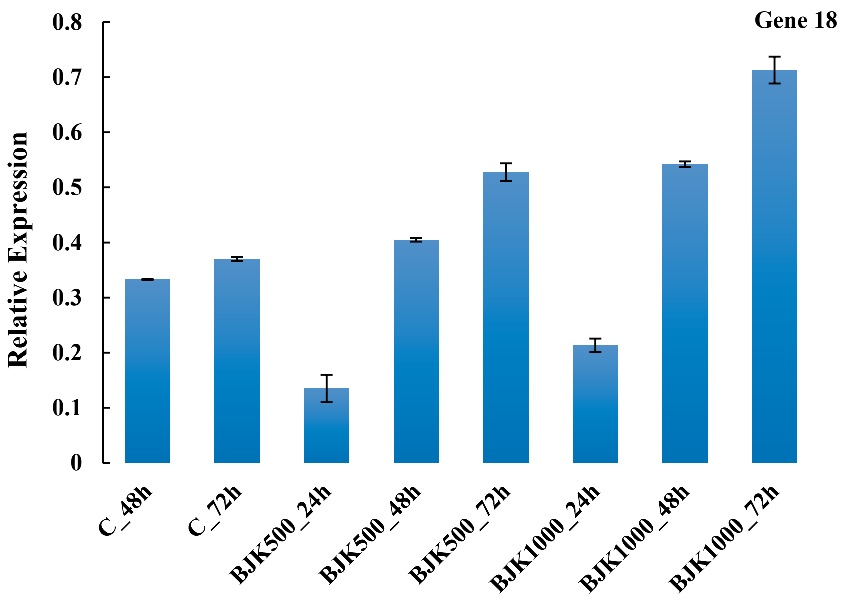
Gene 18
Relative Expression
0.8
0.7
0.6
0.5
0.4
0.3
0.2
0.1
0
C_48h
C_72h
BJK500_24h
BJK500_48h
BJK500_72h
BJK1000_24h
BJK1000_48h
BJK1000_72h

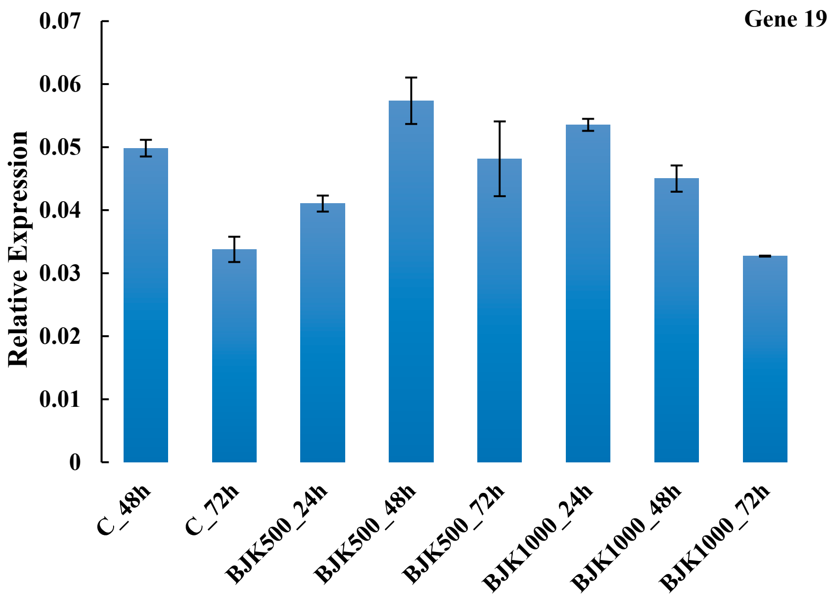
Gene 19
Relative Expression
0.07
0.06
0.05
0.04
0.03
0.02
0.01
0
C_48h
C_72h
BJK500_24h
BJK500_48h
BJK500_72h
BJK1000_24h
BJK1000_48h
BJK1000_72h

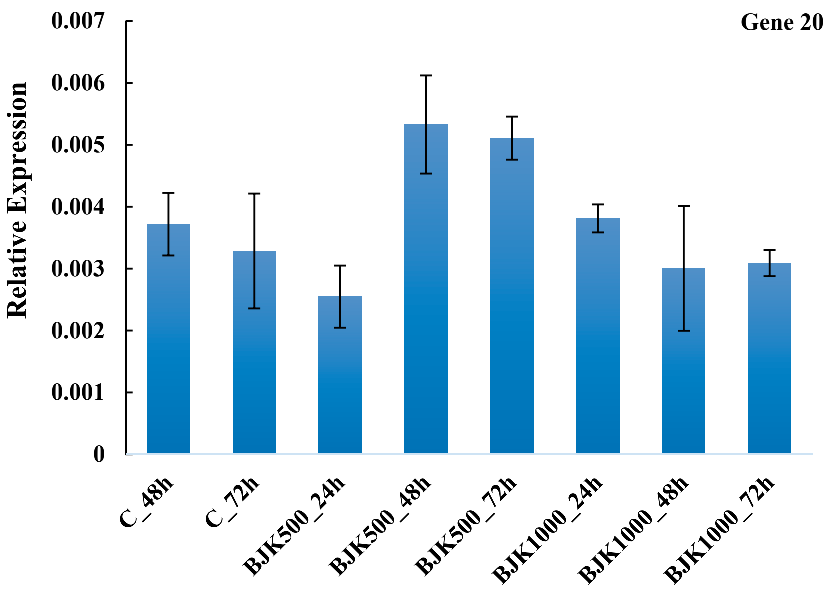
Gene 20
Relative Expression
0.007
0.006
0.005
0.004
0.003
0.002
0.001
0
C_48h
C_72h
BJK500_24h
BJK500_48h
BJK500_72h
BJK1000_24h
BJK1000_48h
BJK1000_72h

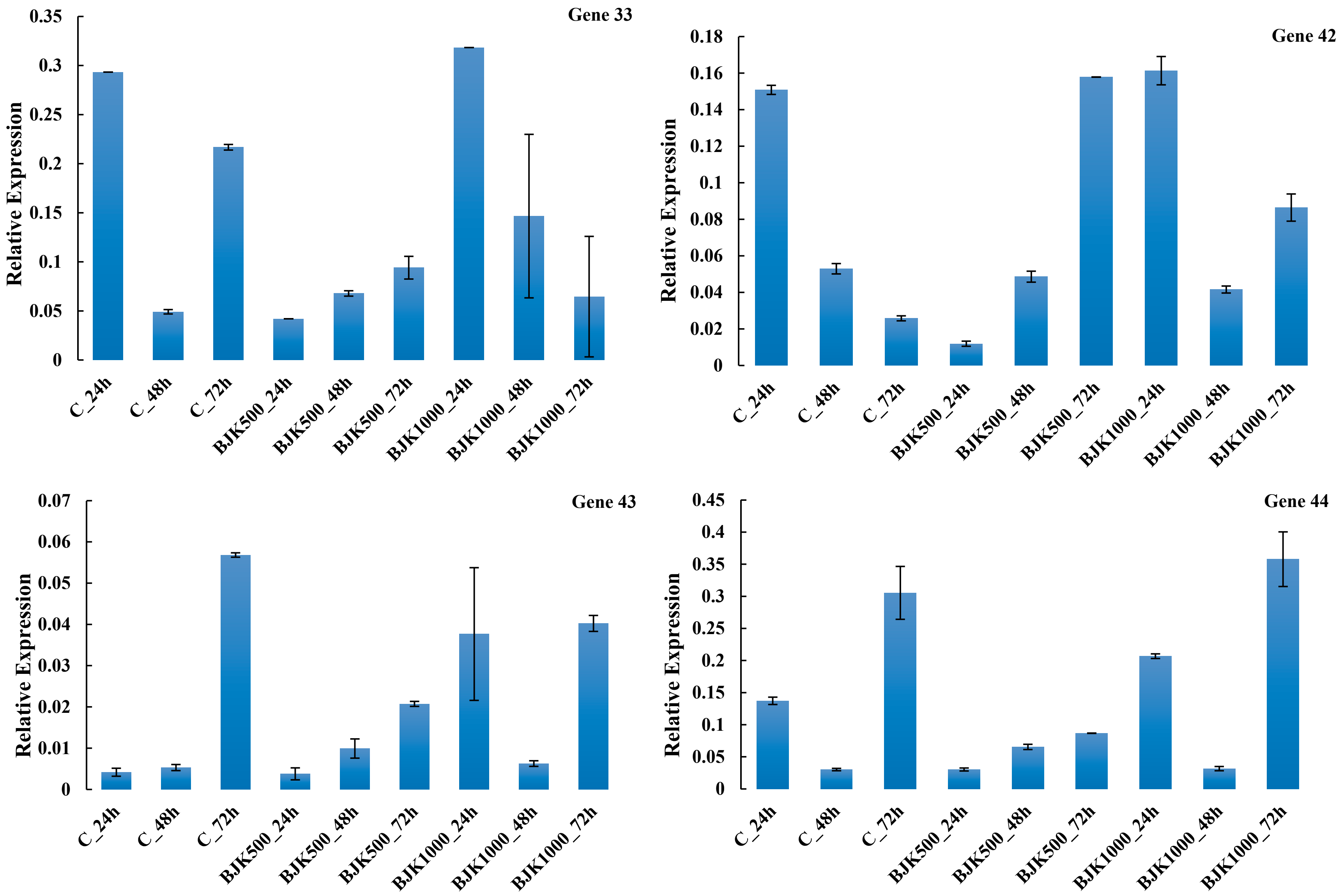
Gene 33
Relative Expression
0.35
0.3
0.25
0.2
0.15
0.1
0.05
0
C_24h
C_48h
C_72h
BJK500_24h
BJK500_48h
BJK500_72h
BJK1000_24h
BJK1000_48h
BJK1000_72h
Gene 42
Relative Expression
0.18
0.16
0.14
0.12
0.1
0.08
0.06
0.04
0.02
0
C_24h
C_48h
C_72h
BJK500_24h
BJK500_48h
BJK500_72h
BJK1000_24h
BJK1000_48h
BJK1000_72h
Gene 43
Relative Expression
0.07
0.06
0.05
0.04
0.03
0.02
0.01
0
C_24h
C_48h
C_72h
BJK500_24h
BJK500_48h
BJK500_72h
BJK1000_24h
BJK1000_48h
BJK1000_72h
Gene 44
Relative Expression
0.45
0.4
0.35
0.3
0.25
0.2
0.15
0.1
0.05
0
C_24h
C_48h
C_72h
BJK500_24h
BJK500_48h
BJK500_72h
BJK1000_24h
BJK1000_48h
BJK1000_72h

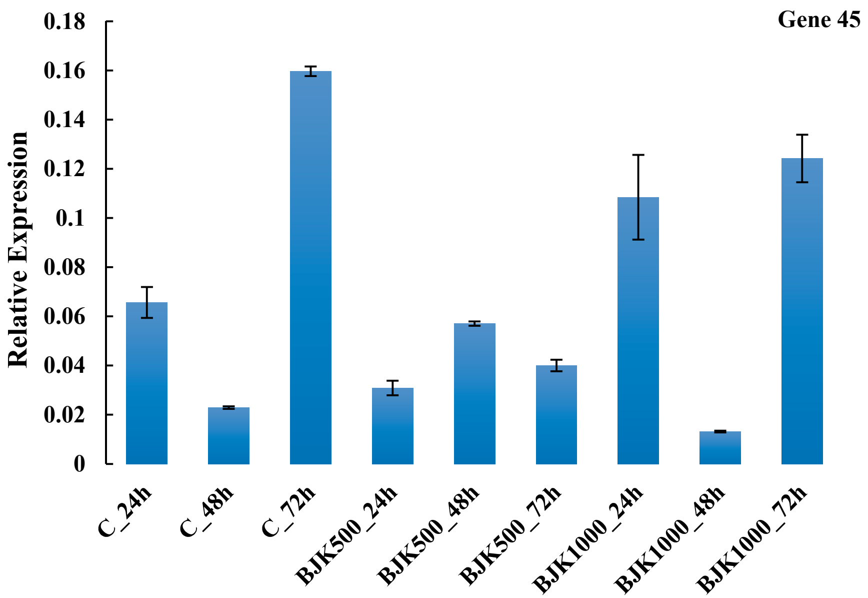
Gene 45
Relative Expression
0.18
0.16
0.14
0.12
0.1
0.08
0.06
0.04
0.02
0
C_24h
C_48h
C_72h
BJK500_24h
BJK500_48h
BJK500_72h
BJK1000_24h
BJK1000_48h
BJK1000_72h

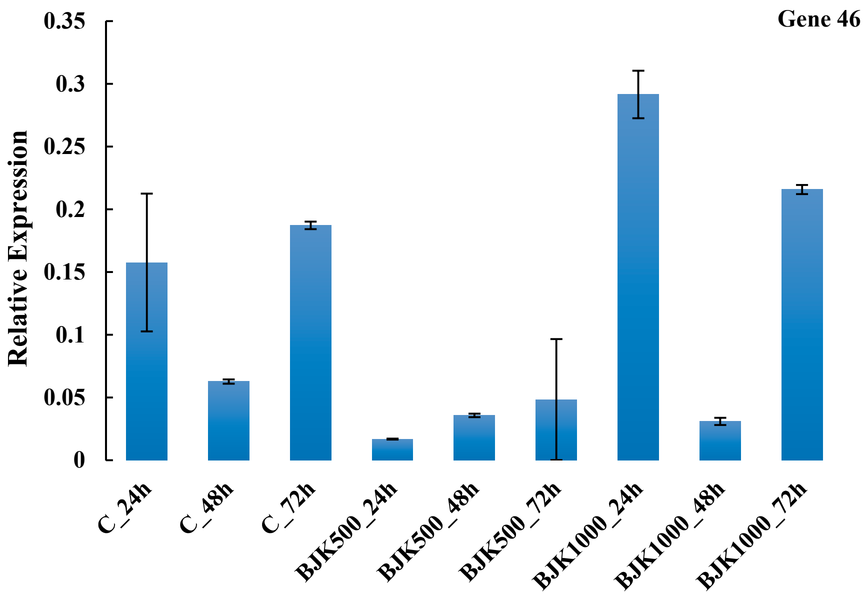
Gene 46
Relative Expression
0.35
0.3
0.25
0.2
0.15
0.1
0.05
0
C_24h
C_48h
C_72h
BJK500_24h
BJK500_48h
BJK500_72h
BJK1000_24h
BJK1000_48h
BJK1000_72h

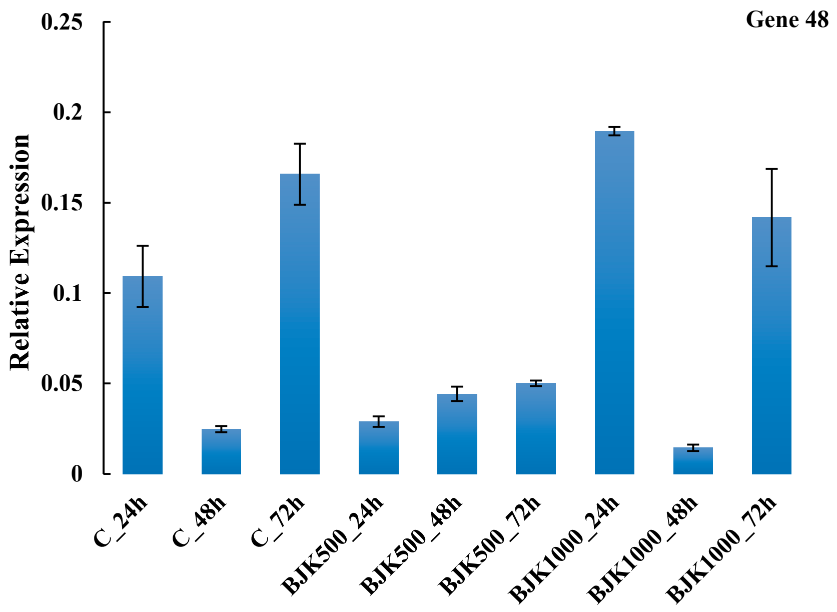
Gene 48
Relative Expression
0.25
0.2
0.15
0.1
0.05
0
C_24h
C_48h
C_72h
BJK500_24h
BJK500_48h
BJK500_72h
BJK1000_24h
BJK1000_48h
BJK1000_72h

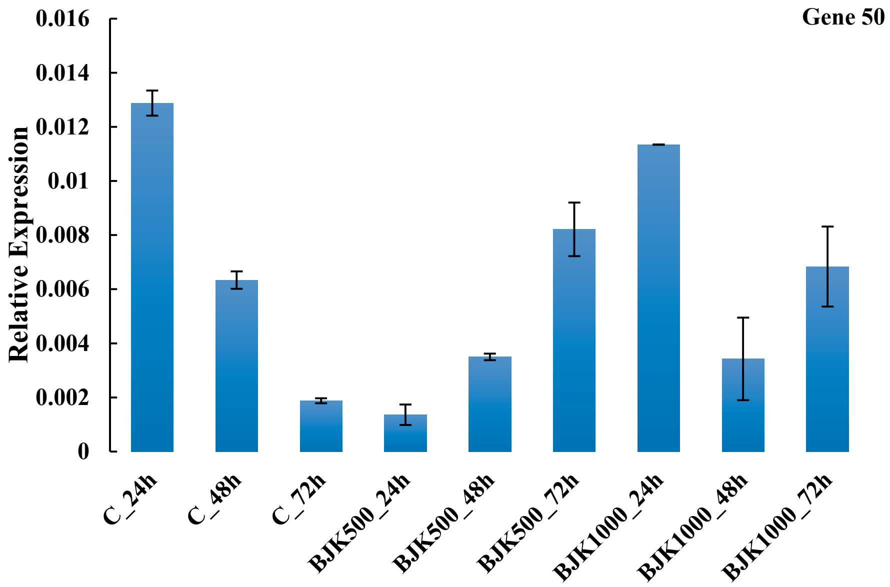
Gene 50
Relative Expression
0.016
0.014
0.012
0.01
0.008
0.006
0.004
0.002
0
C_24h
C_48h
C_72h
BJK500_24h
BJK500_48h
BJK500_72h
BJK1000_24h
BJK1000_48h
BJK1000_72h

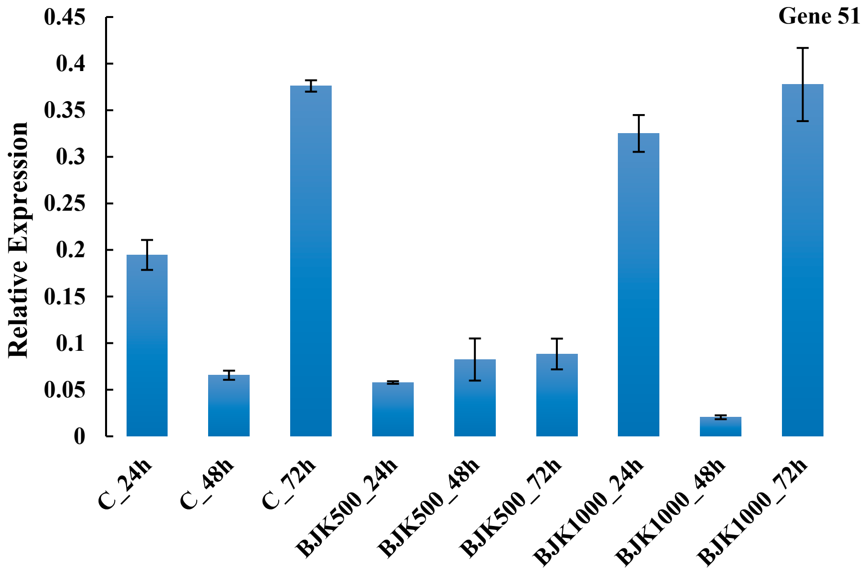
Gene 51
Relative Expression
0.45
0.4
0.35
0.3
0.25
0.2
0.15
0.1
0.05
0
C_24h
C_48h
C_72h
BJK500_24h
BJK500_48h
BJK500_72h
BJK1000_24h
BJK1000_48h
BJK1000_72h

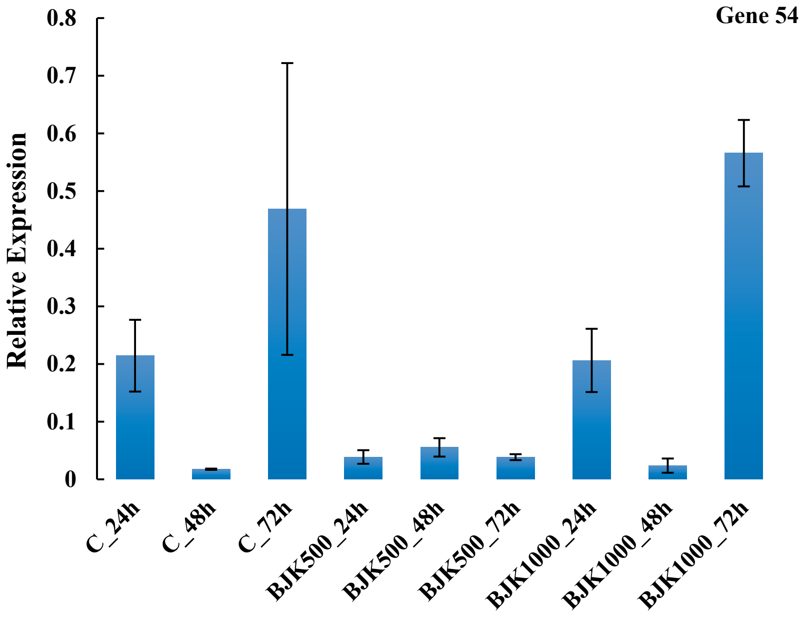
Gene 54
Relative Expression
0.8
0.7
0.6
0.5
0.4
0.3
0.2
0.1
0
C_24h
C_48h
C_72h
BJK500_24h
BJK500_48h
BJK500_72h
BJK1000_24h
BJK1000_48h
BJK1000_72h

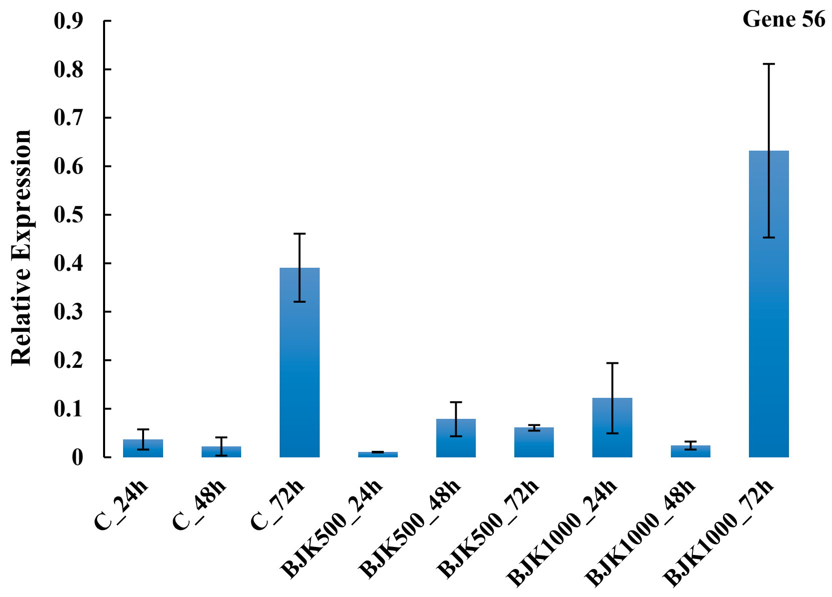

Figure S1. Relative expression of genes in BLACKJAK untreated and treated sugar beet in three time exposures. BJK: BLACKJAK, 500 and 1000 stand for 1.0 mg C L^{-1} and 0.5 mg C L^{-1} BLACKJAK, respectively. Gene codes refer to the name of genes in Table 1.
